# Providing HIV care in the aftermath of Kenya's post-election violence Medecins Sans Frontieres' lessons learned January – March 2008

**DOI:** 10.1186/1752-1505-2-15

**Published:** 2008-12-04

**Authors:** Tony Reid, Ian van Engelgem, Barbara Telfer, Marcel Manzi

**Affiliations:** 1 MSF Brussels, rue Dupre 94, Brussels 1090, Belgium; 2 Former medical coordinator, MSF Kenya, Belgian Technical Cooperation, 41 rue Depute Kayuku – Kiyovu, BP 6089, Kigali, Rwanda; 3 Former epidemiologist, MSF Kenya, Medicos Sem Fronteiras, Belgica-Maputo, Avenida Agostinho Neto N1024, Maputo, Moçambique; 4 FUCHIA Data Manager, MSF Luxembourg, rue de Gasperich 68, L-1617, Luxembourg

## Abstract

Kenya's post-election violence in early 2008 created considerable problems for health services, and in particular, those providing HIV care. It was feared that the disruptions in services would lead to widespread treatment interruption. MSF had been working in the Kibera slum for 10 years and was providing antiretroviral therapy to 1800 patients when the violence broke out. MSF responded to the crisis in a number of ways and managed to keep HIV services going. Treatment interruption was less than expected, and MSF profited from a number of "lessons learned" that could be applied to similar contexts where a stable situation suddenly deteriorates.

## Background

Following the disputed presidential election in Kenya in December 2007, widespread violence broke out resulting in an estimated 1500 people killed and 600,000 displaced from their homes.[1] Although problems had occurred during previous elections, this level of violence was unprecedented in Kenya, a country regarded as a model of democracy and stability. The rapid escalation of events caught many services off guard, and health care was significantly disrupted.

The violence was particularly severe in Kibera, a large slum near Nairobi where Medecins Sans Frontieres (MSF) has been operating three primary health care centers. Medical services in these areas were rapidly affected and there was concern that patients with HIV, in particular, would be unable to obtain their medications, resulting in treatment interruption. The problem was compounded since many patients and health care staff had returned to their home villages to vote, and were prevented from returning to Nairobi due to the violence.

MSF's three clinics in Kibera had been providing comprehensive primary care in addition to full HIV services, including Highly Active AntiRetroviral Therapy (HAART) for several years. By the end of 2007, 5200 patients were enrolled in the HIV program and 1800 were on HAART. [2]

This report describes MSF's response to the challenges of providing HIV services in Kibera slum during the post-election violence. The experience produced some lessons learned that could apply to other contexts that are generally stable but where violence or disorder may be anticipated.

### Setting

Kibera is home to approximately 800,000 people, many of whom are transient and without regular employment. There are very limited municipal services such as sewage and electricity. MSF has been working there for ten years and at the time of the election was operating three health centers; two provided full primary care services with a comprehensive HIV program (Kibera South and Silanga Health Centres) while a third (Gatwekera) offered HIV and TB services only. Patients were initiated on HAART at these clinics and returned for regular follow up at intervals corresponding to their disease status and treatment maturity. Health promotion to increase treatment literacy was a key component.

Once the election results were announced, violence broke out in Kibera with people being beaten, houses burned and police responding with some force. Several people were killed in the ensuing weeks. The fighting continued off and on for most of January and then, as negotiations were announced between President Mwai Kibaki and Opposition Leader Raila Odinga, violence diminished in February and was largely over by the end of March when a power-sharing arrangement between the two leaders was announced. Against this background, MSF struggled to maintain services in Kibera.

### Challenges for the MSF program

The violence in Kibera varied daily, especially in January, making the planning of services a constant challenge. The riots targeted members of the Kikuyu tribe, and since about one third of the clinics' staff were Kikuyu, they were unable to enter Kibera. This created the dual problem of trying to adequately staff the clinics with less well-trained staff, and finding suitable jobs for "displaced" staff.

Normally, the clinics' caseload was a mixture of primary care problems and a comprehensive HIV program. Suddenly, with the violence, there were many cases of acute trauma, (machete wounds, severe beatings), sexual violence and burns from house fires. The workload changed dramatically to managing acutely injured patients.

With the disrupted services, routine data collection and monitoring were compromised. Normally, patients receiving HIV drugs had their appointments and treatment regimes recorded on a special database called FUCHIA. FUCHIA was used to identify patients who had missed appointments and who needed to be traced. However, during the crisis, program monitoring with FUCHIA broke down and patients who had missed appointments could not always be identified. Patient tracing activities were also interrupted, due to insecurity and reduced staff numbers.

During the peaks of violence, Silanga and Gatwekera were not able to open, so their patients came to Kibera South Health Center seeking medication. However, their clinical records were available only in the closed health centers, and their appointment cards did not record clinical data, so it was difficult to be sure of their treatment regimes. Patients who were caught "up country" faced similar difficulties if they went to health centers nearest their homes – they carried no clinical information for the treating clinician.

### MSF responses

Fortunately, MSF's experience in other contexts, and the history of violence during previous elections, meant that the team had an Emergency Preparedness Plan in place. It included clear lines of communication between the various clinics, community members, staff and headquarters. Contacts in government and other NGOs had been brought up to date. Extra stocks of HIV and other drugs/materials were brought in. There was a plan for triage for a sudden influx of acutely ill/traumatized patients. Importantly, many patients on HAART who were due for follow up appointments in January were given an extra supply of medications to carry them over the holiday and election period.

Given the fluctuating level of violence, the Head of Mission assessed security each day using the community contacts and decided which clinics and services could operate safely. Even during the worst days of rioting, MSF was able to keep at least one clinic open. Within the clinics, the triage system was put in place to deal with the sudden influx of trauma victims. Regular primary care services were reduced, and HAART initiation was temporarily halted. A simple emergency monitoring system was rapidly introduced to capture essential data on the number and types of consultations to assist with service delivery planning.

The problems of program monitoring using FUCHIA were rectified to restore the ability to identify patients who had missed appointments. As a temporary measure, clinical and treatment summaries were generated from FUCHIA and given to patients as a record in case they couldn't return for appointments in the future, or needed to seek care at a clinic elsewhere in Kenya.

Then, patient appointment cards were upgraded to "passports" that included current HIV treatment regimens, problems with side effects, and phone numbers of the clinics where patients and clinicians could call for advice.

Perhaps the most ambitious plan was to start a call centre with a toll-free hot line for people on HIV and TB treatment, and other treating clinicians, to provide advice when patients could not return to their usual clinic. MSF had no experience with this, but managed to obtain technical support from a local cell phone company. The center was installed at MSF headquarters and many of the staff who could not work in Kibera were quickly trained to manage calls on the hotline. The hotline was running by Jan 21 and was advertised first in a national newspaper and later on the radio and through flyers and posters distributed countrywide by the Kenyan Red Cross, the Ministry of Health (MoH) and the National Alliance of Churches. In addition to advertising the hotline, communications advised patients on treatment for HIV to attend the nearest clinic to obtain drugs and to adhere to their normal pill regimen as best as possible. The MoH later became involved and planned to use the call center as a clearing house for all HIV services in the country.

### Treatment interruption results

Despite the initial problems with data collection, these were corrected and the FUCHIA database was restored. In order to assess the number of patients who might have experienced treatment interruption of HAART, we analyzed the number of patients who were delayed for their follow up appointment for the months of January to March 2008, and compared them to the same three months of 2007. As Table 1 shows, there was a rise in delayed appointments in January 2008, approximately double that seen in January 2007, while the results from February and March showed no difference.

**Table 1:** Comparison of HAART Consultations January to March, 2007–2008

**Kibera**						
	**2007**			**2008**		

	**Jan**	**Feb**	**March**	**Jan**	**Feb**	**March**

Consultations	1196	1035	1226	1237	1146	1223

**Delayed**	**76 (6.4%)**	69 (6.7%)	51 (4.2%)	**162 (13.1%)**	82 (7.2%)	51 (4.2%)

**Lost To Follow Up**	23 (1.9%)	26 (2.5%)	41 (3.3%)	36 (2.9%)	41 (3.6%)	45 (3.7%)

**Transferred out**	3	1	8	3	7	2

These figures were obtained from FUCHIA following its restoration.

"Consultations" were all HAART consultations.

"Delayed" were defined as missing an assigned appointment by more than 7 days.

"Lost to Follow Up" was a standard FUCHIA outcome for missing an appointment by more than one month.

"Transferred out", also standard FUCHIA outcome, meant a formal transfer to another health center.

In addition, as patients were seen in follow up, clinicians noted, anecdotally, that quite a few had obtained medications from alternate sources and so that even though their appointments had been delayed, they had not suffered a treatment interruption. A manual chart review was carried out for all patients who were delayed in their return appointment, checking for actual breaks in medication continuity. Unfortunately, this information was not available for all patients from the charts. As per Table 2, note that at least a third (and possibly more) reported not missing their medication. Patients reported obtaining extra medications from various sources: shared between spouses, or friends, or they obtained them from a nearby clinic. This would imply that treatment literacy messages regarding avoiding treatment interruption were strong enough to encourage patients to be creative when their appointments were delayed.

**Table 2:** Manual chart assessment of HAART treatment interruption

	Not indicated	Missed pills	Did not miss	File lost, etc
**Silanga**	22	22	26	5

**KSHC**	16	16	30	10

**Gatwekera**	31	46	43	7

**Total**	69 (25%)	84 (31%)	99 (36%)	22 (8%)

A manual chart review was carried out by MSF staff of all patients who had "delayed appointments".

Unfortunately, the hotline did not function as well as planned. Based on reports of approximately 950 calls over 3 months, only 10% were for HAART treatment advice, 21% were for other HIV issues and the rest were for other medical or non-medical issues. It is not known how many HIV patients followed the advice given via the media to attend their nearest clinic for drugs. One problem was that people could only access the toll free hotline if they used the dedicated cell phone company. Many people, using the other main cell phone provider in Kenya, were unable to access the toll free hotline. In addition, there was limited support from the phone company, and the staff had some difficulty in adapting to answering calls related to general health, or unrelated issues. Consequently, while it was difficult to measure the effect of the call center on HIV treatment access, the impact appeared to be limited. Had the call center been established prior to the crisis, with better planning, it might have been better utilized.

## Conclusion

Despite considerable challenges, MSF was able to keep medical services and HAART treatment functioning in the midst of post-election violence in Kibera, Nairobi. A number of patients experienced delays in their follow up appointments, but the delays were confined to the month of January and by February appointments were back to normal. Many of those patients with delayed appointments still managed to continue treatment. These results were likely due to a combination of factors: a relatively short crisis, with order being restored quickly, MSF's preparedness and commitment to continued care, good patient literacy and strong community support throughout the crisis.

## Lessons learned

Providing ongoing HAART treatment in resource-poor contexts is always a challenge, but Kenya's experience demonstrated an apparently stable situation can deteriorate quite quickly. Advanced planning for such contingencies and the ability to adapt a program rapidly will reduce the extent of treatment interruption. Thus, we recommend that in contexts that appear to be stable,

▪ Develop an Emergency Preparedness Plan that covers the essential elements of an HIV program should the medical system be destabilized. This should be updated in times of anticipated problems. Emergency preparedness planning should include discussions with relevant MoH agencies and other service delivery providers.

▪ Establish a communications structure with close ties to the community, facilitating daily situation assessments and permiting a program to be tailored to the level of security and need.

▪ Develop a simple, emergency data management system that can continue to record clinical information during the crisis and that contains the essential information for ongoing care.

▪ Use a "patient passport" that contains current and previous HAART treatment regimens, and side effects, so that care can be obtained at other health centers. The card could also include a telephone number of the home clinic so patients or clinicians could call for information.

▪ In times of anticipated instability, consider extending the supply of medication for patients. Similarly, increase stocks of medications in the health centers to anticipate interruptions of drug supply.

▪ If a call center is planned, consider a modification based on our experience. Health centers could establish a regular phone "hot line" that would be answered by staff both during regular times and emergencies, to provide treatment advice for patients and other treating clinicians. Having the phone number printed on the "Patient Passport" and including its purpose in treatment literacy would increase its effectiveness.

## Ethics

This report is based on routinely-collected data from the MSF program in Nairobi and as such does not require formal ethics approval, according the MSF Ethics Review Board.

## Competing interests

None of the authors has any competing interests, financial or otherwise. All are, or were at the time, employees of MSF.

## Authors' contributions

TR conceived the idea, collated the information from the field, and was the principal author. IvE was medical coordinator during the time of the violence and contributed to the history of the events as well as some data analysis. BT was the epidemiologist with MSF in Nairobi at the time and contributed to the history of the project as well as the data management during the conflict. MM carried out the data analysis from FUCHIA. All authors contributed to and approved the final version of the article

## Funding

This report was funded from regular MSF program budget. No additional funds were obtained.
